# Incidence and risk factors of retinopathy of prematurity in artificial reproductive technology conceived preterm infants in China

**DOI:** 10.1186/s40942-026-00859-6

**Published:** 2026-04-17

**Authors:** Shufen Chen, Shuhui Chen, Yuzhen Zhu, Yuxuan Lai, Guihua Xu

**Affiliations:** 1 The First School of Clinical Medicine, Guangdong Medical University, Zhanjiang, Guangdong 524023 China; 2 Department of Ophthalmology, Huizhou Mingkang Ophthalmology Hospital, Huizhou City, Guangdong Province People’s Republic Of China; 3 Department of Ophthalmology, Huizhou Central People’s Hospital, Huizhou City, Guangdong Province People’s Republic of China

**Keywords:** Retinopathy of prematurity, Assisted reproductive technology, Natural conception, Incidence, Preterm infants

## Abstract

**Background:**

To evaluate the incidence and associated risk factors of retinopathy of prematurity (ROP) in preterm infants conceived through assisted reproductive technology (ART) in China.

**Methods:**

We conducted a retrospective analysis of preterm infants who were hospitalized between August 2019 and December 2024. Infants were divided into two groups based on mode of conception: ART-conceived and naturally conceived. The incidence of ROP was compared between the groups. Clinical data were collected for both the infants and their mothers.

**Results:**

A total of 1,019 preterm infants were included, comprising 195 ART-conceived and 824 naturally conceived infants. ROP was diagnosed in 33 ART-conceived (16.9%) and 136 naturally conceived infants (16.5%), with no statistically significant difference between the two groups (*P* = 0.888). Similarly, no significant difference was observed in ROP staging (*P* = 0.228). Multivariable logistic regression analysis showed that, in the ART-conceived group, greater gestational age (OR: 0.618) and a history of antenatal corticosteroid use (OR: 0.278) were protective factors, whereas apnea of prematurity (OR: 3.170) increased the risk of ROP. Similarly, in the naturally conceived group, greater gestational age (OR: 0.840) was also a protective factor, while apnea of prematurity (OR: 1.666) increased the risk of ROP.

**Conclusion:**

The incidence of ROP did not differ significantly between ART-conceived and naturally conceived preterm infants. In ART-conceived infants, higher gestational age and a history of antenatal corticosteroid use were protective factors, whereas apnea of prematurity increased the risk of ROP.

## Background

Retinopathy of prematurity (ROP) is a vasoproliferative disorder of the developing retina that predominantly affects preterm and low birth weight infants [1–3]. Although advances in neonatal care have significantly improved the survival of premature infants, these improvements may inadvertently contribute to an increased incidence of ROP [4]. Currently, ROP is the most common cause of neonatal blindness globally, with a growing burden particularly evident in middle-income countries across Asia [5].

Low gestational age and low birth weight are the most well-established risk factors for ROP [6]. In recent years, the widespread adoption of assisted reproductive technology (ART) has been associated with increased rates of preterm birth and low birth weight when compared to natural conception [7, 8]. These ART-related perinatal outcomes suggest a potential link between ART and ROP. However, existing literature on whether ART is an independent risk factor for ROP remains inconclusive. For instance, Barker et al. [9] found no significant difference in ROP incidence between ART and non-ART groups, even among multiple-birth infants. Conversely, Chan et al. [10] reported an association between ART and severe ROP. Nonetheless, many of these studies are now outdated, with more than a decade having passed since data collection. Given the rapid evolution and refinement of ART techniques, it remains unclear whether the current generation of ART-conceived infants remains at increased risk for ROP. To our knowledge, apart from one article discussing whether artificial conception is a risk factor for ROP in very low birth weight (less than 1500 g) preterm infants in China [11], no other related studies have been conducted in the region.

Furthermore, ROP is increasingly recognized as a multifactorial disease influenced by both neonatal and maternal factors. These include maternal gestational diabetes, hypertensive disorders of pregnancy, prenatal infections, mode of delivery, multiple gestations, and neonatal complications such as bronchopulmonary dysplasia, apnea of prematurity, and respiratory distress syndrome [6, 12]. However, most previous ROP studies have primarily focused on general neonatal and maternal risk factors, while relatively limited data are available regarding risk profiles specifically in ART-conceived preterm infants.

Therefore, the purpose of the present study was to evaluate the incidence of ROP in infants conceived by assisted technology in China, compare it with that in naturally conceived preterm infants, and analyze the associated maternal and neonatal risk factors contributing to ROP in preterm infants conceived through ART.

## Methods

Premature infants hospitalized in the Neonatal Care Center of Dongguan People’s Hospital from August 2019 to December 2024 were enrolled in this retrospective study. Infants were categorized into two groups: naturally conceived and artificially conceived. The study complied with the principles of the Declaration of Helsinki, and the research protocol was approved by the Medical Ethics Committee of Dongguan People’s Hospital. Written informed consent was obtained from the parents of all participating infants. In accordance with the national guidelines for ROP screening in China [13], infants meeting the following criteria were enrolled: (1) preterm and low-birth-weight infants with birth weight < 2000 g or gestational age < 32 weeks; (2) infants with severe systemic illness or a clear history of prolonged oxygen therapy, for whom neonatologists recommended fundus examination. Exclusion criteria were: (1) infants in the naturally conceived group whose mothers had a history of infertility and conceived following pharmacological treatment; (2) infants who were re-hospitalized during the neonatal period, which could lead to duplicate enrollment; and (3) cases with incomplete clinical data.

Ophthalmic examinations were performed by ophthalmologists using an indirect ophthalmoscope. The first screening was conducted 4–6 weeks after birth, and ROP was staged according to the International Classification of ROP, based on the revised 2021 criteria [14]. Clinical data collected from infants included sex, gestational age, birth weight, history of mechanical ventilation and continuous positive airway pressure, intraventricular hemorrhage, phototherapy, patent ductus arteriosus, congenital heart disease, apnea of prematurity, bronchopulmonary dysplasia, respiratory distress syndrome, thrombocytopenia, elevated liver enzymes, fetal anemia, fetal blood transfusion, and ICU monitoring. Maternal clinical data included maternal age, history of gestational diabetes, gestational hypertension, prenatal infection, preeclampsia, prenatal steroid use, mode of delivery (vaginal or cesarean), and type of pregnancy (singleton or multiple).

### Statistical analysis

Data analysis was performed using SPSS (version 13.0; SPSS Inc., Chicago, IL, USA). The incidence of ROP was calculated and compared between the two groups. Differences in ROP incidence rates were evaluated using the chi-square test. Group comparisons of clinical parameters were conducted with independent t-tests and χ² tests. Variables that showed statistically significant differences in the comparison between the ROP and non-ROP groups within the ART-conceived or naturally conceived cohorts were included in the multivariable logistic regression analysis to identify independent risk factors for ROP. A *p*-value < 0.05 was considered statistically significant.

## Results

**Fig. 1:**
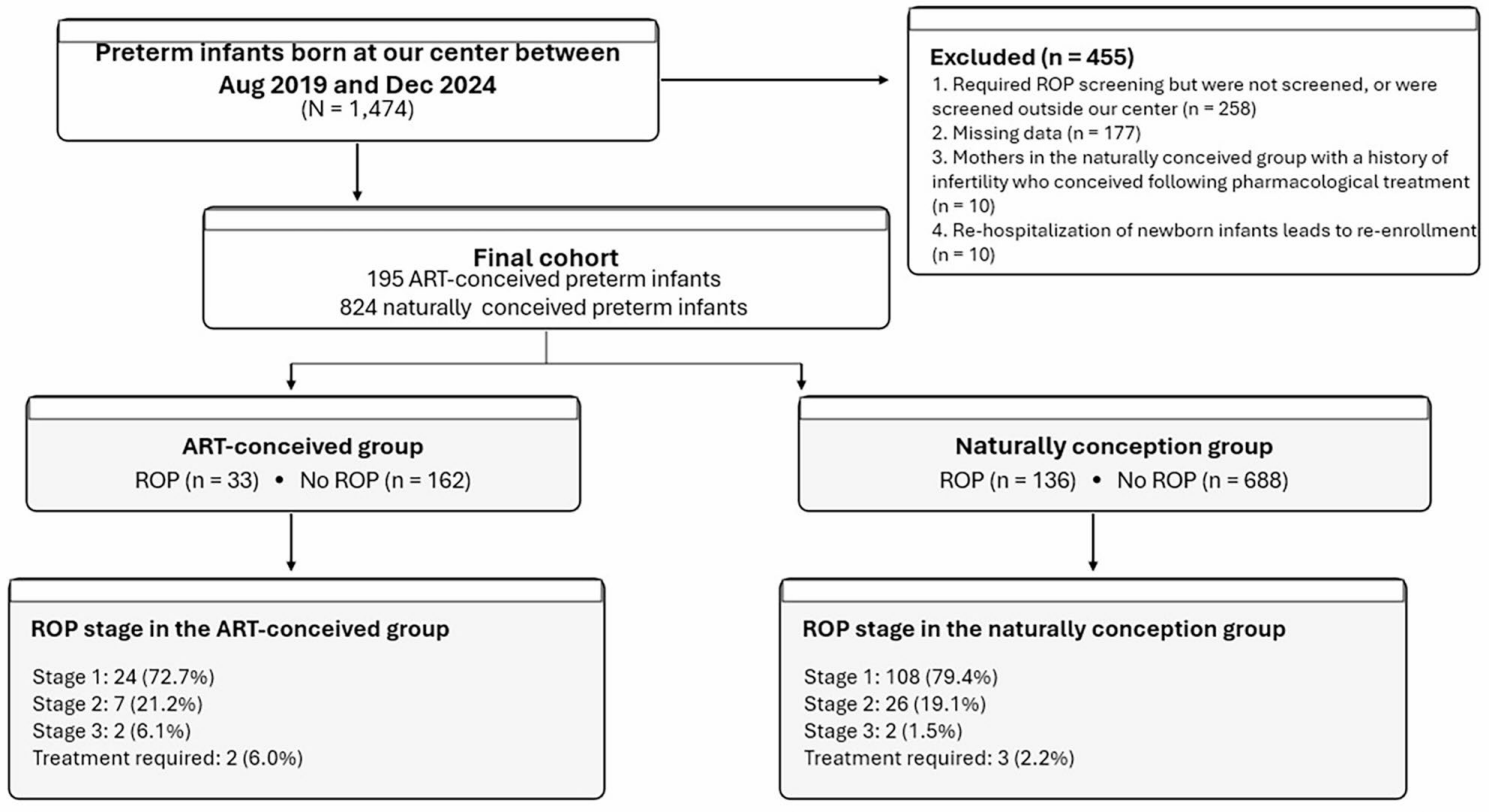
Flowchart of the study population

Initially, a total of 1,474 preterm infants were screened. The screening process is shown in Fig. 1. Of these, 455 were excluded for the following reasons: (1) infants who required ROP screening but were either not screened or screened at another hospital (258 cases); (2) incomplete clinical data (177 cases); (3) mothers in the naturally conceived group with a history of infertility who conceived following pharmacological treatment (10 cases); and (4) infants who were re-hospitalized during the neonatal period, which could lead to duplicate enrollment (10 cases). Ultimately, 1,019 infants were included in the analysis, comprising 195 preterm infants conceived via ART and 824 naturally conceived preterm infants.

The incidence of ROP was 16.9% in the ART group and 16.5% in the non-ART group, showing no statistically significant difference (*P* = 0.888). The total incidence across both groups was 16.6%. Among ART-conceived infants with ROP (*n* = 33), 24 (72.7%) were Stage 1, 7 (21.2%) were Stage 2, and 2 (6.1%) were Stage 3; none progressed to Stage 4 or 5. Among naturally conceived infants with ROP (*n* = 136), 108 (79.4%) were Stage 1, 26 (19.1%) were Stage 2, and 2 (1.5%) were Stage 3, with no Stage 4 or 5 disease. The distribution of ROP stages did not differ significantly between groups (*P* = 0.228). Regarding treatment, intervention was required in 6.1% (*n* = 2) of ART-conceived infants and 2.2% (*n* = 3) of naturally conceived infants with ROP, a difference that was not statistically significant (*P* = 0.252) (Table 1).

**Table 1:** Incidence and staging of ROP in neonates of natural and ART

Variables	ART group (*n* = 195)	Natural group (*n* = 824)	*P* Value
ROP n (%)	33 (16.9%)	136 (16.5%)	0.888
ROP Stage			0.228
Stage1, n (%)	24 (72.7%)	108 (79.4%)	
Stage 2, n (%)	7 (21.2%)	26 (19.1%)	
Stage 3, n (%)	2 (6.1%)	2 (1.5%)	
Need treatment, n (%)	2 (6.1%)	3 (2.2%)	0.252

ART, artificial reproductive technology; ROP, retinopathy of prematurity

As shown in Table 2, common factors associated with ROP in both ART-conceived and naturally conceived preterm infants included: lower gestational age, low birth weight, use of mechanical ventilation and longer duration of oxygen therapy (including mechanical ventilation and continuous positive airway pressure), respiratory diseases (including bronchopulmonary dysplasia, apnea of prematurity, and respiratory distress syndrome), anemia of prematurity, and blood transfusion of prematurity. Additionally, the data indicate that for preterm infants with ROP conceived via ART, patent ductus arteriosus and congenital heart disease may be additional associated factors (*P* < 0.05). For naturally conceived preterm infants with ROP, maternal prenatal infection, preeclampsia, and antenatal corticosteroid use may be additional associated factors (*P* < 0.05).

**Table 2:** Characteristics of neonates born by ART and natural

Variables	ART with ROP (33)	ART without ROP (162)	*P* Value	Natural group with ROP (136)	Natural group without ROP (688)	*P* Value
Neonatal factors						
Gestational Age (week)	29.5 ± 5.5	33.5 ± 2.3	<0.001	31.5 ± 2.8	33.5 ± 2.3	<0.001
Birth weight (g)	1482.4 ± 455.4	1958.3 ± 494.1	<0.001	1571.9 ± 561.1	1982.6 ± 554.9	<0.001
Gender, Male (%)	26(78.8%)	100(62.5%)	0.062	71(52.2%)	382(55.5%)	0.477
Mechanical ventilation, N (%)	30(90.9%)	111(68.5%)	0.009	115(84.6%)	481(69.9%)	<0.001
Days on mechanical ventilation	15.2 ± 14.8	4.5 ± 6.6	<0.001	12 ± 14.5	4.6 ± 7.2	<0.001
CPAP, N (%)	23(69.7%)	83(51.2%)	0.052	85 (62.5%)	395 (57.4%)	0.272
Days on CPAP	7.2 ± 10	2.5 ± 5.5	<0.001	4.4 ± 6.3	2.2 ± 4.9	<0.001
Intraventricular hemorrhage, N (%)	3(9.0%)	17(10.5%)	1.000	13(9.5%)	41(6.0%)	0.121
Phototherapy, N (%)	31(93.9%)	157(96.9%)	0.746	129 (94.8%)	668 (97.1%)	0.281
Patent ductus arteriosus, N (%)	10(30.3%)	25(15.4%)	0.042	27 (19.9%)	98 (14.2%)	0.096
Congenital heart disease, N (%)	10(30.3%)	25(15.4%)	0.042	24 (17.6%)	125 (18.2%)	0.885
Apnea of prematurity, N (%)	17(51.5%)	22(13.6%)	<0.001	47 (34.6%)	103 (15.0%)	<0.001
Bronchopulmonary dysplasia, N (%)	6(18.2%)	1(1%)	<0.001	20 (14.7%)	12 (1.7%)	<0.001
Respiratory distress syndrome, N (%)	28(84.8%)	105(64.8%)	0.024	113 (83.1%)	450 (65.4%)	<0.001
Thrombocytopenia, N (%)	2(6%)	7(4.3%)	1.000	14 (10.3%)	53 (7.7%)	0.312
Elevated liver enzyme levels, N (%)	11(33.3%)	46(28.4%)	0.570	51 (37.5%)	267 (38.8%)	0.775
Anemia, N (%)	19(57.6%)	50(30.9%)	0.003	81 (59.6%)	198 (28.8)	<0.001
Blood transfusion, N (%)	18(54.5%)	50(30.9%)	0.009	80 (58.8%)	184 (26.7%)	<0.001
ICU, N (%)	13 (39.4%)	66(45.1%)	0.550	72 (52.9%)	304 (44.2%)	0.061
Pregnant woman factor						
Childbearing age	33.5 ± 4.1	32.8 ± 4.3	0.370	31.2 ± 4.8	30.6 ± 5.2	0.235
History of gestational diabetes, N (%)	4(12.1%)	46(28.4%)	0.051	41 (30.1%)	183 (26.6%)	0.395
History of gestational hypertension, N (%)	5(15.2%)	23(14.2%)	1.000	26 (19.1%)	91 (13.2%)	0.072
Maternal prenatal infection, N (%)	18 (54.5%)	74 (45.7%)	0.352	90 (66.2%)	376 (54.7%)	0.013
Preeclampsia, N (%)	3 (9%)	26 (16%)	0.450	33 (24.3%)	116 (16.9%)	0.04
History of antenatal corticosteroid use, N (%)	23(69.7%)	110 (67.9%)	0.840	98 (72.1%)	428 (62.2%)	0.029
Type of delivery, CS, N (%)	28(84.8%)	126(77.8%)	0.364	81 (59.6%)	425 (61.8%)	0.628
Multiple births, N (%)	19 (57.6%)	104 (64.2%)	0.472	19 (14.0%)	93 (13.5%)	0.888

ART, artificial reproductive technology; ROP, retinopathy of prematurity; CPAP, continuous positive airway pressure; ICU, intensive care unit; CS, cesarean section

Logistic regression analysis of 195 ART-conceived preterm infants showed that greater gestational age (*P* = 0.045, OR: 0.618, 95% CI: 0.386–0.990) and a history of antenatal corticosteroid use (*P* = 0.039, OR: 0.278, 95% CI: 0.082–0.939) were protective factors, whereas apnea of prematurity (*P* = 0.037, OR: 3.170, 95% CI: 1.072–9.371) increased the risk of ROP (Table 3).

**Table 3:** Multivariate regression analysis of influencing factors for ROP in artificially conceived preterm infants

Variables	*p* value	Odds Ratio	95%CI
Gestational Age (week)	0.045*	0.618	0.386–0.990
Birth weight (g)	0.408	0.416	0.052–3.321
Mechanical ventilation, N (%)	0.945	1.092	0.090-13.314
Days on mechanical ventilation	0.582	1.022	0.946–1.104
Days on CPAP	0.189	1.044	0.979–1.112
Patent ductus arteriosus, N (%)	0.772	0.819	0.212–3.161
Congenital heart disease, N (%)	0.217	2.046	0.657–6.376
Apnea of prematurity, N (%)	0.037*	3.170	1.072–9.371
Bronchopulmonary dysplasia, N (%)	0.663	1.901	0.105–34.298
Respiratory distress syndrome, N (%)	0.875	0.838	0.093–7.557
Anemia, N (%)	0.240	0.399	0.086–1.848
Blood transfusion, N (%)	0.148	0.309	0.063–1.515
Maternal prenatal infection, N (%)	0.323	1.698	0.595–4.846
Preeclampsia, N (%)	0.979	1.022	0.210–4.970
History of antenatal corticosteroid use, N (%)	0.039*	0.278	0.082–0.939

CI, confidence interval; CPAP, continuous positive airway pressure; * *P* < 0.05

Logistic regression analysis of 824 naturally conceived preterm infants showed that greater gestational age (*P* = 0.020, OR: 0.840, 95% CI: 0.725–0.973) was a protective factor, whereas apnea of prematurity (*P* = 0.035, OR: 1.666, 95% CI: 1.038–2.674) increased the risk of ROP (Table 4).

**Table 4:** Multivariate Regression Analysis of Influencing Factors for ROP in Naturally Conceived Preterm Infants

Variables	*p* value	Odds Ratio	95%CI
Gestational Age (week)	0.020*	0.840	0.725–0.973
Birth weight (g)	0.477	0.798	0.428–1.487
Mechanical ventilation, N (%)	0.518	0.698	0.234–2.078
Days on mechanical ventilation	0.307	1.015	0.986–1.045
Days on CPAP	0.942	0.999	0.966–1.033
Patent ductus arteriosus, N (%)	0.126	0.629	0.348–1.138
Congenital heart disease, N (%)	0.900	0.967	0.572–1.634
Apnea of prematurity, N (%)	0.035*	1.666	1.038–2.674
Bronchopulmonary dysplasia, N (%)	0.107	2.339	0.832–6.572
Respiratory distress syndrome, N (%)	0.903	1.069	0.368–3.105
Anemia, N (%)	0.450	1.247	0.703–2.214
Blood transfusion, N (%)	0.238	1.413	0.796–2.509
Maternal prenatal infection, N (%)	0.165	1.351	0.883–2.067
Preeclampsia, N (%)	0.150	1.456	0.873–2.430
History of antenatal corticosteroid use, N (%)	0.627	0.889	0.555–1.426

CI, confidence interval; CPAP, continuous positive airway pressure; * *P* < 0.05

## Discussion

Our study found no significant difference in the incidence of ROP between preterm infants conceived via ART and those conceived naturally. In ART-conceived infants, greater gestational age and a history of antenatal corticosteroid use were identified as protective factors, while apnea of prematurity increased the risk of ROP. Across both groups, related influencing factors included low gestational age, low birth weight, use of mechanical ventilation, prolonged oxygen therapy, respiratory diseases (including apnea of prematurity, bronchopulmonary dysplasia, and respiratory distress syndrome), anemia of prematurity, and a history of blood transfusion.

With the rising prevalence of ART and growing concern about ROP in preterm infants, clarifying the relationship between the two is important. Our findings are in line with several recent reports. Trifonova et al. (2018) [4] conducted a systematic review and found no statistical correlation between ART and ROP, while Gao et al. [1] reported a positive association, particularly with stage 3 ROP. Wu et al. (2018) [11], studying very low birth weight infants (< 1500 g) in China, identified in vitro fertilization as an independent risk factor for ROP. However, most studies included in the above meta-analyses were published before 2017, with some dating back decades, raising concerns about methodological bias. Moreover, the infants in Wu et al.’s study were enrolled more than ten years ago, when ART techniques were less mature and neonatal care standards were lower, potentially leading to higher complication rates in ART-conceived preterm infants. Earlier studies suggested that in vitro manipulations, cryopreservation of gametes or embryos, and culture conditions could adversely affect ART outcomes [15], but these influences have diminished with technological advances. Two recent studies consistently reported no correlation between ART and ROP [16, 17]. Alsammahi et al. (2021) [16] analyzed 229 preterm infants (mean gestational age 29.35 weeks) and found no difference in ROP incidence between naturally conceived and ART-conceived groups, despite ART infants being predominantly multiples. Similarly, Vardhelli et al. (2022) [17] examined 759 preterm infants (26–31 weeks of gestation) and found no difference in the incidence of ROP. In our cohort, the total incidence of ROP in premature infants was 16.6%. The reported incidence of any-stage ROP in preterm infants varies across countries, with rates of 10% to 34% in Europe and the United States [18], and 11.9% to 26.0% in China [2], which was consistent with our findings.

Our results confirm that greater gestational age is a protective factor, aligning with the well-established understanding that lower gestational age is a risk factor for ROP [2]. In addition, our study identified a history of antenatal corticosteroid use as a protective factor in ART-conceived infants. This finding may be explained by the well-recognized clinical benefits of antenatal corticosteroids, which have been shown to significantly reduce neonatal respiratory distress syndrome and mortality, with benefits that substantially outweigh the potential risks [19, 20]. Moreover, a 2018 systematic review and meta-analysis reported that antenatal corticosteroid use was associated with a reduced risk of both ROP occurrence and progression to severe ROP [21].

Multivariable regression analysis showed that apnea of prematurity increased the risk of ROP in both the ART-conceived and naturally conceived groups. The main adverse effect of apnea of prematurity on ROP lies in its induction of recurrent hypoxia, oxygen saturation fluctuations, and bradycardia, which can disrupt the normal development of immature retinal vessels and thereby increase the risk of ROP onset and progression [22]. In a study by Wu et al. [11] involving 504 very low birth weight infants (< 1500 g), apnea of prematurity was identified as an independent perinatal risk factor for ROP. Ramirez et al. [23] also reported that apnea of prematurity was a risk factor for ROP progression. Mechanistically, apnea episodes are often followed by hypoxia and subsequent repeated fluctuations in oxygen tension due to reoxygenation or supplemental oxygen support. This hypoxia–reoxygenation process may induce oxidative stress and affect angiogenesis-related pathways such as vascular endothelial growth factor (VEGF), thereby disturbing retinal vascularization. In preterm infants with limited antioxidant capacity, this effect may be even more pronounced and may therefore contribute to the development of ROP [22].

Our study also showed that use of mechanical ventilation or longer duration of oxygen therapy and the presence of respiratory diseases were commonly related factors for ROP in both ART-conceived and naturally conceived preterm infants, which aligns with previous research [11]. One possible explanation is that oxidative stress is believed to be associated with ROP [19], while both mechanical ventilation and respiratory diseases are associated with increased oxidative stress [24]. Furthermore, fetal anemia and a history of blood transfusion were identified as common related factors for ROP in both groups, consistent with earlier studies [16]. A possible reason is that fetal hemoglobin, the primary oxygen transport protein, releases oxygen at lower tissue oxygen levels than adult hemoglobin. However, transfusions for anemia in preterm infants introduce adult hemoglobin, which is more prone to inducing oxidative stress reactions [19].

Our study has several limitations. First, it was conducted at a single tertiary hospital, which might limit generalizability to the broader Chinese population. Second, some infants were excluded because they were not screened at our institution or had incomplete data, which may have introduced selection bias. Third, because the number of Stage 3 or treatment-requiring ROP cases was small, we did not perform subgroup analyses based on disease severity. Finally, detailed information on the specific ART modality was unavailable in some cases, which precluded further subgroup analyses according to ART type. Future studies with larger sample sizes are needed to validate our findings.

## Conclusions

In conclusion, this study found no significant difference in ROP incidence between ART-conceived and naturally conceived preterm infants. In ART-conceived infants, greater gestational age and a history of antenatal corticosteroid use were protective factors, whereas apnea of prematurity increased ROP risk. These findings suggest that while ART itself may not directly increase the incidence of ROP, pregnancy and perinatal-related factors play a critical role in disease development.

## Data Availability

The data presented in this study are only available on request from the corresponding author due to the author’s workplace policy on medical data.
